# The Role of TNF-α and Anti-TNF-α Agents during Preconception, Pregnancy, and Breastfeeding

**DOI:** 10.3390/ijms22062922

**Published:** 2021-03-13

**Authors:** Katarzyna Romanowska-Próchnicka, Anna Felis-Giemza, Marzena Olesińska, Piotr Wojdasiewicz, Agnieszka Paradowska-Gorycka, Dariusz Szukiewicz

**Affiliations:** 1 Department of Biophysics and Human Physiology, Faculty of Health Sciences, Warsaw Medical University, 02-091 Warsaw, Poland; kromanowska@wum.edu.pl (K.R.-P.); pwojdasiewicz@gmail.com (P.W.); dszukiewicz@wum.edu.pl (D.S.); 2 Department of Connective Tissue Diseases, National Institute of Geriatrics, Rheumatology and Rehabilitation, 02-637 Warsaw, Poland; marzena.olesinska@vp.pl; 3 Department of Molecular Biology, National Institute of Geriatrics, Rheumatology and Rehabilitation, 02-637 Warsaw, Poland; agnieszka.paradowska-gorycka@spartanska.pl

**Keywords:** TNF-α, anti-TNF-α, placental transfer, procreation, fertility, pregnancy, breastfeeding

## Abstract

Tumor necrosis factor-alpha (TNF-α) is a multifunctional Th1 cytokine and one of the most important inflammatory cytokines. In pregnancy, TNF-α influences hormone synthesis, placental architecture, and embryonic development. It was also shown that increased levels of TNF-α are associated with pregnancy loss and preeclampsia. Increased TNF-α levels in complicated pregnancy draw attention to trophoblast biology, especially migratory activity, syncytialisation, and endocrine function. Additionally, elevated TNF-α levels may affect the maternal-fetal relationship by altering the secretory profile of placental immunomodulatory factors, which in turn affects maternal immune cells. There is growing evidence that metabolic/pro-inflammatory cytokines can program early placental functions and growth in the first trimester of pregnancy. Furthermore, early pregnancy placenta has a direct impact on fetal development and maternal immune system diseases that release inflammatory (e.g., TNF-α) and immunomodulatory factors, such as chronic inflammatory rheumatic, gastroenterological, or dermatological diseases, and may result in an abnormal release of cytokines and chemokines in syncytiotrophoblasts. Pregnancy poses a challenge in the treatment of chronic disease in patients who plan to have children. The activity of the disease, the impact of pregnancy on the course of the disease, and the safety of pharmacotherapy, including anti-rheumatic agents, in pregnancy should be considered.

## 1. The Role of TNF-α in Pregnancy

Tumor necrosis factor-alpha (TNF-α) is a multifunctional Th1 cytokine and one of the most important inflammatory cytokines. It is produced by macrophages during inflammation and can also be activated by the endotoxin lipopolysaccharide (LPS). TNF-α controls the growth of normal and neoplastic cells, has a profound effect on the expression of genes related to cell differentiation, and influences the function of different cells.

TNF-α has been shown to play a role in the pathogenesis of various inflammatory diseases, such as rheumatoid arthritis, Crohn’s disease, spondyloarthropathies, psoriasis, systemic lupus erythematosus (SLE) or antiphospholipid syndrome, as well as in atherosclerosis, diabetes mellitus, and metabolic syndrome [1]. In pregnancy, TNF-α influences hormone synthesis, placental architecture, and embryonic development [2]. It was also shown that increased levels of TNF-α are associated with pregnancy loss [3] and preeclampsia [4].

The mother-fetus interaction requires a perfect synergy between the maternal immune system and inflammatory responses, which allows for the immune adaptation of the fetus and, at the same time, maintains immune responses at a level that confers protection from potential infections.

TH1 and TH2 cytokine homeostasis allows for embryo implantation and normal pregnancy outcomes. At the early stages of normal pregnancy, Th1 pro-inflammatory cytokines are necessary for the stimulation of new vessels for successful embryo implantation [5]. However, prolonged exposure to Th1 cytokines may result in a cell-mediated immune response, which is harmful to the fetus and may cause spontaneous abortion [6].

It was noted at the beginning of the twenty-first century that the expression of Th2 cytokines, the anti-inflammatory interleukin IL10 in particular [7,8,9], was increased in normal pregnancy, whereas women after pregnancy loss presented a higher expression of Th1 cytokines [10]. The expression of cytokines, as well as the resulting immune response, may also be the result of cytokine gene polymorphism. Thus, cytokine gene polymorphisms can potentially contribute to the risk of recurrent miscarriage, especially gene polymorphisms associated with pro-inflammatory cytokines such as TNF-α, Il-1, Il-6, and IFNG [11,12,13,14,15]. TNF-α may directly promote tissue damage in pregnancy, as suggested by in vitro studies where TNF-α activated maternal monocytes bound to LFA-1 on placental syncytiotrophoblasts and induced apoptosis [16]. Maternal blood levels of TNF-α during pregnancy increase in direct proportion to the stage of pregnancy, as well as in the postpartum period [17,18].

Increased TNF-α levels have been shown to be associated with a number of adverse effects, such as gestational hypertension and gestational diabetes mellitus (GDM) [18,19]. Increased TNF-α levels in complicated pregnancy draw attention to trophoblast biology, especially migratory activity, syncytialisation, and endocrine function [20,21].

Additionally, elevated TNF-α levels may affect the maternal-fetal relationship by altering the secretory profile of placental immunomodulatory factors, which in turn affects maternal immune cells. Indeed, literature data suggest that trophoblast-derived factors can induce the differentiation of peripheral blood monocytes into macrophages [22], as well as increasing recruitment and differentiation of inducible regulatory T cells (Treg) [23].

To conclude, the placenta is an immunomodulatory organ that regulates both local (embryo implantation) and systemic immune responses [24]. The highly differentiated syncytiotrophoblast is an integral part of the placenta, and the barrier connecting the placental villi with the maternal blood directly exposes the fetus to maternal cytokines.

Diseases that release inflammatory (e.g., TNF-α) and immunomodulatory factors, such as chronic inflammatory rheumatic, gastroenterological, or dermatological diseases, may result in the abnormal release of cytokines and chemokines in syncytiotrophoblasts. There is growing evidence that metabolic/pro-inflammatory cytokines can program early placental functions and growth in the first trimester of pregnancy [25]. Furthermore, early pregnancy placenta has a direct impact on fetal development and the maternal immune system [26]. Homeostasis of pro-and anti-inflammatory cytokines is the subject of interest of many scientific studies. Maintaining this fragile balance between pro- and anti-inflammatory factors is crucial for successful implantation and normal pregnancy outcomes. Recent studies have shown a shift in the cytokine profile of the human placenta in the first trimester of pregnancy towards increased levels of GM-CSF, CCL5, and IL10 in response to increased maternal TNF-α levels, while IL-6 and IL-8 remain unchanged [27]. This may be a protective mechanism of the human placenta in the first trimester of pregnancy to maintain trophoblast function and suppress inflammatory processes in the intervillous space to improve isolation from the inflammatory processes in maternal blood. However, this issue requires much research, especially in larger groups of patients.

## 2. Rheumatic and Gastroenterological Diseases in Pregnancy

A total of 80% of patients with chronic inflammatory rheumatic, gastroenterological and dermatological diseases are at a reproductive age.

Pregnancy poses a challenge in the treatment of chronic disease in patients who plan to have children. The activity of the disease, the impact of pregnancy on the course of the disease, and the safety of pharmacotherapy, including anti-rheumatic agents, in pregnancy, should be considered.

Sustained clinical remission of the underlying disease is the ideal time for conception. A recently published meta-analysis in pregnant patients with rheumatoid arthritis (RA), which was based on 10 different studies (a total of 237 pregnant and 135 puerperal patients), showed that the activity of RA decreased in an average of 60% (40% to 90%) of pregnant patients, whereas exacerbation was observed in 46.7% (39% to 70%) of puerperal patients [28,29].

The majority of retrospective studies showed no effects of RA activity on pregnancy. It should be noted that further studies (at least three) have shown that infants born to mothers with RA present with lower birth weight, although the majority are within the normal range [30,31,32]. A relationship was found between RA exacerbation during pregnancy and the low birth weight of the child. Data on psoriatic arthritis are very limited and are based on single, prospective studies where exacerbations were reported for approximately 22%, unchanged psoriatic arthritis (PsA) activity for 50%, and reduced symptoms for 28% of pregnant women [33].

For seronegative spondyloarthritis (SpA), literature reviews (mainly retrospective studies) have shown highly varied, controversial, and ambiguous data [34]. About 80% of pregnant patients with SpA present with stable disease activity or worsening of symptoms, which often improve during the third trimester. However, deterioration is observed after delivery (6–12 weeks after delivery) [35,36]. Zbiden et al. showed that 78% of women with axial spondyloarthritis (axSpA) experienced disease exacerbation in pregnancy, especially in the second trimester [37]. This risk is increased in patients who are not in remission at conception, those with elevated acute-phase protein (CRP), especially at 20 weeks gestation (second trimester), and women who discontinued anti-TNF-α therapy once pregnancy was confirmed [38,39].

Discontinuation of TNF-α inhibitor treatment itself causes a 3.08-fold increase in the risk of disease exacerbation throughout pregnancy in women with axSpA. Interestingly, patients without preconception exposure to anti-TNF-α also experienced high persistent disease activity from the preconception period until postpartum [40].

The Visual Analog Scale (VAS) indicated more severe pain in pregnant patients with ankylosing spondylitis (AS) compared to those with RA [29]. It should also be emphasized that standard methods for pain measurement are of no use in pregnancy because the non-specific lumbosacral pain may worsen, especially in the third trimester [29].

Studies on inflammatory bowel diseases (IBDs) deliver much more epidemiological data.

A large prospective multicenter study (PIANO), which included over 1475 pregnant women with IBD, showed a higher rate of ulcerative colitis activity compared to Crohn’s disease activity during pregnancy [41,42]. A hypothesis of overlapping immune pathways, including the production of pro-inflammatory cytokines by the placenta [43], was proposed as a potential explanation [43].

Additionally, the risk of exacerbation during pregnancy was 20% for Crohn’s disease and 33% for ulcerative colitis among women with IBDs who had remission of their underlying disease at conception [42,43].

A meta-analysis in 1475 patients with IBD (PIANO) showed a strong correlation between disease activity during pregnancy and disease activity in general [42,43]. Disease exacerbation affects 46% to 55% of women who conceived during active IBD compared to 23% to 29% of pregnancies started during remission. The rates of IBD relapse during pregnancy are generally similar to those for non-pregnant women [44]. Exacerbation during pregnancy and puerperium may be associated with discontinuation of disease-modifying therapy [45,46]. Therefore, optimized care, patient awareness, appropriate disease treatment, and control prior to conception helps maintain IBD remission throughout pregnancy.

## 3. Anti-TNF-α Agents

The anti-TNF-α group was introduced on the market more than two decades ago. For over twenty years, growing evidence has accumulated that these agents can be safely used during pregnancy and breastfeeding and that they do not cause genetic defects in the fetus.

At the time of the introduction of anti-TNF-α drugs (more than 20 years ago), the summaries of product characteristics (SmPC) did not allow for their use during preconception, pregnancy, and breastfeeding. Furthermore, evidence for the lack of effects on the fetus was missing. Observational studies based on individual case reports, case series, and, over time, cohort observations or registry analyses gradually appeared, confirming the safety of individual anti-TNF-α agents, and allowing the classification of this group of drugs as FDA safety category B. (Table 1.)

The majority of gastroenterological, dermatological, and rheumatological societies allow for the use of anti-TNF-α treatment only in the event of exacerbation of rheumatological diseases, inflammatory bowel diseases, or psoriasis.

Anti-TNF-α agents are used in the following rheumatological diseases: rheumatoid arthritis, non-radiographic seronegative spondyloarthritis, ankylosing spondylitis (AS), psoriatic arthritis, as well as in gastroenterology in inflammatory bowel diseases (Crohn’s disease, ulcerative colitis). and in dermatology in plaque psoriasis.

There are five anti-TNF-α drugs available on the market (by order of registration: infliximab, etanercept, adalimumab, certolizumab, golimumab). The first three already have their biosimilars. (a) Infliximab (IFX), a chimeric anti-TNF-α monoclonal antibody composed of human constant regions and murine variable regions of kappa light chains and IgG heavy chains [47]. It has an affinity to the soluble and transmembrane forms of human TNF-α but does not bind to β lymphotoxin (TNF-β). Infliximab causes the loss of TNF-α biological activity by binding to TNF-α in plasma and tissues. It is used for RA, PsA, AS, psoriasis, and IBD [48]. (b) Etanercept (ETA), a soluble recombinant TNF-Fc complex composed of extracellular fragments of two soluble CHO cell-derived TNF-α receptors linked to a human IgG Fc fragment [47]. The mechanism of action of the drug involves competitive inhibition of TNF-α binding to its surface receptors and prevention of a TNF-α-mediated cellular response. It is used in RA, PsA, AS, SpA, and psoriasis [49]. (c) Adalimumab (ADA), a monoclonal IgG1 anti-TNF-α antibody identical to the natural human antibody [47]. The drug is expressed in Chinese hamster ovary cells. Adalimumab binds to TNF-α and neutralizes its biological activity by blocking the p55 and p75 receptors on the cell surface. Additionally, adalimumab regulates changes in the levels of cell adhesion molecules, which are responsible for leukocyte migration. The drug is used for AR, PsA, SpA, AS, enthesitis-related arthritis (ERA), plaque psoriasis, purulent inflammation of the apocrine sweat glands, IBD, juvenile idiopathic arthritis (JIA), and uveitis [50]. (d) Certolizumab pegol (CZP) is is a polyethylene glycol (PEG)-conjugated recombinant humanized Fab’ fragment of antitumor necrosis factor-alpha (TNF-α) antibody, expressed in *Escherichia coli* cells. It neutralizes both the membrane and the soluble form of human TNF-α in a dose-dependent manner. Incubation of monocytes with certolizumab resulted in dose-dependent inhibition of lipopolysaccharide (LPS)-induced TNF-α and interleukin-1β (IL-1β) production in human monocytes. The drug is used for AR, PsA, SpA, AS, and plaque psoriasis [51]. (e) Golimumab (GOL), a human IgG1κ monoclonal antibody produced by the murine hybridoma cell line by recombinant DNA technology. The binding of human TNF-α by golimumab neutralizes TNF-α-induced cell surface expression of the E-selectin adhesion molecule, vascular intercellular adhesion molecule (VCAM-1), and the intercellular adhesion molecule (ICAM-1) of endothelial cells. Golimumab inhibits the secretion of IL-6, IL-8, and TNF-α-induced granulocyte colony-stimulating factor (GM-CSF) in vitro. It is used for AR, PsA, SpA, AS, and JIA [52].

**Figure 1:**
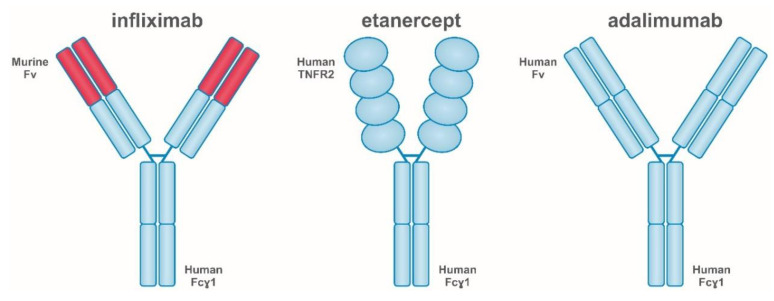

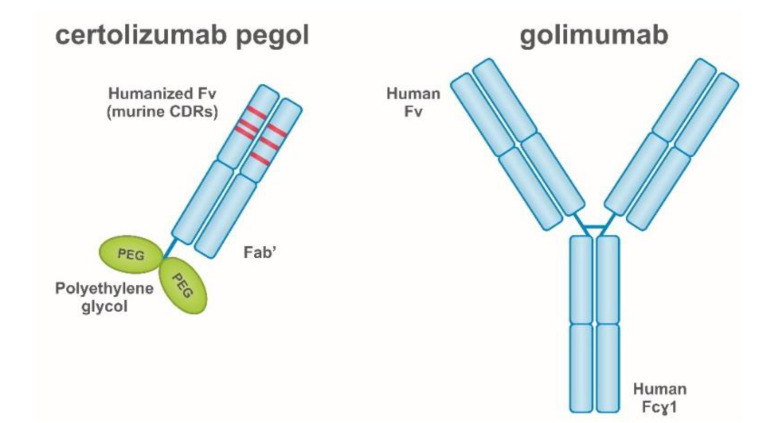
Structure and nomenclature of tumor necrosis factor-alpha (TNF-α) inhibitors.

Infliximab, adalimumab, and golimumab are IgG1 antibodies capable of binding the complement and the Fc-binding receptor. The hinge region of certolizumab is modified and covalently attached to 2 cross-linked 20 kDa chains of polyethylene glycol to increase the in vivo solubility and half-life (Figure 1).

## 4. Fertility and Biological Therapies

### 4.1. Men

While the role of TNF-α in male spermatogenesis is still under investigation, published scientific reports suggest that TNF-α plays a critical role in spermatogenesis and maintaining the blood-testis barrier [53,54,55].

Normal spermatogenesis involves the apoptosis of some of the developing germ cells. It is estimated that up to 75% of the hypothetical sperm count is lost due to apoptosis in early spermatogenesis [53]. The reason for this cell loss in the testes is to maintain an optimal germ cell/Sertoli cell ratio. Sertoli cells are essential for the maturation of germ cells [54].

TNF-α signals through two transmembrane receptors, TNFR1 and TNFR2. TNFR1 contains the cytoplasmic death domain. TNFR1 activates the caspase cascade through the death domain, thereby leading to cell death. However, TNFR1 also mediates effects that promote cell survival by activation of transcription factors, such as NF-kB and AP-1, which can induce genes involved in the suppression of apoptosis. In human testes, TNF-α inhibits gametogenic cell apoptosis, is produced by germ cells, and acts as a paracrine cytokine by binding to both Sertoli and Leydig cells [53,54,55]. TNF-α plays an important role in regulating apoptosis of gametogenic cells, which can be blocked by an anti-TNF-α agent, by promoting their survival [56].

A 54% reduction in sperm production was found in a murine model of TNF-related-to-apoptosis-inducing ligand (TRAIL) deficiency, where TNF-α signaling was impaired [57]. Despite its undeniable role in spermatogenesis, high levels of TNF-α may also be harmful. If its levels are not regulated, it may lead to complications (orchitis) [58,59]. The effect of inhibition of TNF-α (anti-TNF-α therapy) on human fertility is poorly understood. While some studies have shown no harmful effects of anti-TNF-α agents on sperm quality, spermatogenesis, anti-sperm antibody production, and testosterone levels [59,60], other studies have shown an increased risk of asthenospermia (decreased sperm motility) [61,62,63] and abnormal sperm morphology [64,65]. The literature on inflammatory rheumatic diseases treated with biologics showed a significant reduction in the number of sperm after the onset of anti-TNF-α treatment [66]. Fertility was assessed in 34 men (at a reproductive age) treated with adalimumab, 9 men treated with etanercept, and 84 men treated with infliximab [47]. There are isolated case reports of decreased semen quality after exposure to anti-TNF-α agents [67]. Reduced sperm motility and abnormal sperm morphology (asthenozoospermia) have also been described in some cases of infliximab-treated AS patients [62]. Other studies in a small group of patients have shown a statistically insignificant relationship between infliximab and reduced sperm mobility or abnormal sperm morphology; however, researchers have also pointed to other potential causative factors, such as alcohol abuse, nicotinism, and eating disorders [62,63,64,65]. Despite the “very low strength of evidence” for the effect of anti-TNF-α agents on male fertility, Semet et al. found in their review study on the effects of these drugs on male fertility that semen cryopreservation and treatment discontinuation for the fear of fertility loss were not necessary [66]. It is worth adding that men who were informed about the possible impact of anti-TNF-α drugs on fertility before treatment onset were much more likely to opt for cryopreservation. Nevertheless, the percentage of men who decided to undergo semen cryopreservation was low (<6%) [60]. Interestingly, summaries of product characteristics for individual anti-TNF-α drugs, which are available on the website of the European Medicines Agency (EMA, www.ema.europa.eu (accessed on 18 February 2021)), only informs that the effect on human fertility has not been studied (ADA, ETA), that it is unknown whether the drug affects fertility (INF), or that it does not affect fertility in animals (GOLI) and humans (CZP) [48,49,50,51,52]. Chronic inflammatory diseases affect sexual function and libido and thus have an indirect impact on fertility. It has been shown that men with IBD are at an increased risk of hypogonadism, decreased libido, difficulty achieving or maintaining an erection, and depression compared to age-matched controls [68,69,70,71]. Patients with AS have problems with a stiff back or even loss of spinal mobility, which is associated with erectile dysfunctions and decreased sexual satisfaction [72]. Hypogonadism is associated with elevated inflammatory markers and the risk of autoimmune diseases in men. In the case of patients with good control of autoimmune inflammatory disease (RA) activity, no restoration of a normal level of total testosterone (eugonadism) has been shown [73,74]. However, most of the available reports on the fertility of men on anti-TNF-α therapy due to inflammatory disease indicate that the treatment is safe and has negligent effects on fertility disorders [47]. However, it is emphasized that better control of inflammatory disease activity (rheumatic or gastrointestinal) is associated with improved semen parameters [75,76,77]. Hence, the 2016 recommendations of the British Society for Rheumatology (BSR) and the British Health Professionals in Rheumatology (BHPR) and the 2020 recommendations of American experts (American College of Rheumatology, ACR) indicate that there is no need to discontinue anti-TNF-α treatment in men who plan to have children [77,78].

### 4.2. Women

Although no increased risk of infertility has been demonstrated in women and men with IBD in clinical remission and without a history of surgery [79], the activity of the disease and the widely understood treatment have an impact on fertility and pregnancy. Drugs used to treat IBDs (biologics, steroids, thiopurines, methotrexate, and mesalazine) do not reduce fertility [80,81,82].

Women with active inflammatory bowel disease may have decreased fertility [83] associated with dyspareunia in the course of severe rectal or pelvic disease, obstruction of the fallopian tube secondary to pelvic adhesions, and ovarian dysfunction associated with chronic disease or nutritional deficiencies [84]. Female patients with rheumatic diseases may have reduced fertility. The median percentage of subfertility is 9% in the general population compared to 25–42% in RA patients [85,86]. It is believed that many factors contribute to impaired fertility. Non-steroidal anti-inflammatory drugs (NSAIDs) and high doses of prednisone (>7.5 mg/day) contribute to prolonged time to pregnancy (TTP) [85]. A retrospective study has shown that treatment with a biological disease-modifying anti-rheumatic drug (bDMARD) at conception may reduce TTP in RA patients [87]. Active disease (RA) is also associated with decreased fertility. TTP > 1 year was found in 67% of women with high disease activity (DAS28-CRP > 5.1) compared to 30% among women in remission (DAS28-CRP 2.6) [85]. Women with RA may experience earlier menopause [88]. Female patients with RA have fewer children, which reflects the personal choices of women with chronic diseases [86]. Unexplained infertility is diagnosed in 41% of RA patients compared to 8–28% in the general population [89].

In conclusion, disease activity, age, and type of treatment (cyclophosphamide) may have a negative impact on fertility in patients with rheumatic diseases [77].

## 5. Placental Transfer of Biologicals and Maternal/Child Safety during Pregnancy and Breastfeeding

In pregnancy, mother-to-fetus transfer of antibodies occurs through the placenta. The transfer occurs through the neonatal Fc receptor (FcRn) and provides immunity to the newborn.

Fetal immunoglobulin (Ig) levels increase steadily from the first days of the second trimester to delivery. Fetal immunoglobulin (Ig) levels increase steadily from the first days of the second trimester until delivery. Most antibodies are transferred in the third trimester. IgG1 is the most efficiently transported Ig subclass.

It has long been known that IgG is the only class of antibodies that is actively transferred from mother to child to provide short-term passive immunity. This specific IgG transport is mediated by the neonatal Fc receptor (FcRn). FcRn is expressed by syncytiotrophoblasts, where it transports IgG from the maternal circulation to the fetal capillaries of the placental villi [90,91,92].

**Figure 2:**
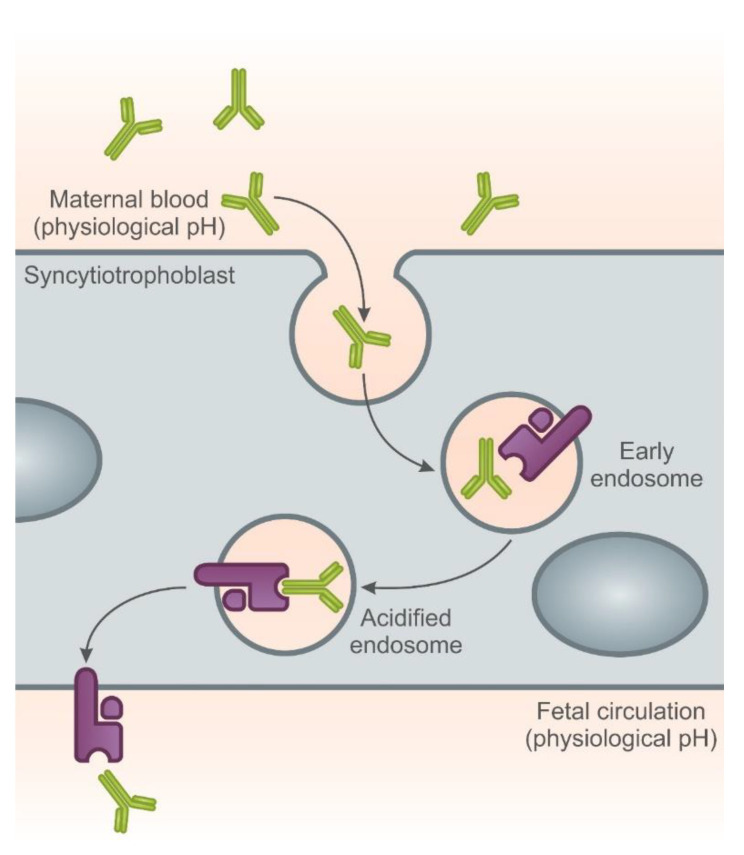
The neonatal Fc receptor (FnRn) mediates the perinatal transfer of IgG. In humans, the neonatal Fc receptor for IgG (FcRn) binds maternal IgG in an acidic environment, transcytoses is across a polarized epithelial-cell barrier and releases a physiological pH. In humans, maternal-fetal IgG transfer occurs across the syncytiotrophoblast of the placenta. FcRn is expressed in the internal vesicles of the syncytiotrophoblast. On acidification in the endosome, FcRn binds to maternal IgG and transcytoses it to the fetal circulation where it is released as physiological pH.

The syncytiotrophoblast absorbs fluid containing maternal IgG into endosomes. The IgG-containing endosome is then gradually acidified, thereby allowing for tight binding between IgG and the Fc receptor (FcRn). The endosome then fuses with the membrane on the fetal side of the syncytiotrophoblast, where the physiological pH promotes the dissociation of the IgG from the FcRn (Figure 2).

IgG and FcRn molecules can also migrate back to the maternal placenta. Such retrograde motion may be a mechanism for FcRn recovery [93].

Certolizumab (CZP) has a unique structure compared to other approved anti-TNF-α drugs. This may prevent fetal exposure to the drug during pregnancy. CZP is a monovalent Fab’ fragment of a humanized monoclonal element conjugated to a polyethylene glycol (PEG) chain, which consequently lacks the Fc region (Figure 1). Other drugs with the structure of monoclonal antibodies (infliximab and adalimumab) and the tumor necrosis factor-α receptor II: IgG Fc fusion protein (etanercept) have an IgG1 Fc region (Figure 1). The binding of the IgG Fc region to the neonatal Fc receptor (FcRn) plays an important role in regulating IgG homeostasis by protecting antibodies from degradation [94,95]. The Fc domain of IgG is also involved in the active mother-to-fetus transport of antibodies across the placenta, which is mediated by FcRn binding. Therefore, it should be expected that the presence or absence of the Fc region will affect the binding of biologicals to FcRn and affect their active transport across the placenta.

## 6. Infliximab

Literature data on placental transfer of infliximab are based on IFX-treated patients with inflammatory bowel disease. Minimal drug transfer in the first trimester, which is a critical period of organogenesis, was confirmed. Mahadevan [96] assessed umbilical cord blood and infliximab levels in the infant for 11 mothers who received infliximab. The levels of the drug in infants ranged between 2.9 and 39.5 μg/mL [96]. All these levels were higher compared to the corresponding time-matched maternal levels and the levels at delivery. Drug levels persisted for 7 months after birth. Similar findings were obtained in a larger multicenter study [97] in patients receiving infliximab, with a median drug level in the cord blood of 5.9 µg/mL [97]. The median infant-maternal drug concentration ratio was 1.97, and the clearance time in the infant was up to 7.3 months. It was only at 12 months of age that no detectable levels of INF were found in children. It can be concluded based on these data that neonatal levels of infliximab depend on maternal drug levels, which in turn depend on the time interval from the last dose. Further studies showed an increase in the concentration of infliximab depending on the stage of pregnancy, with an increase of 4.2 μg/mL per trimester [98]. It has been proposed to discontinue infliximab in the second and third trimesters to limit intrauterine exposure of the fetus.

The safety of infliximab has been confirmed in many studies over two decades [41,42,43,44,45,46,99,100,101,102,103,104,105].

A thorough analysis of the data indicates that a large study was conducted in a French cohort with over 1457 pregnant patients on infliximab. Risk assessment showed a higher risk of maternal complications (OR 1.49), mainly maternal infections (OR 1.31). These infections mostly resolved spontaneously, with less than 50% of cases requiring antibiotic therapy. It should also be added that continuation of anti-TNF-α therapy after 24 weeks did not increase the risk of maternal complications, and that discontinuation of treatment and maternal exposure to relapse of the inflammatory disease in pregnancy was a higher risk. The decision as to whether and when to discontinue infliximab, as well as other biologics, remains controversial.

The safety of the child should also be considered in the case of infliximab therapy.

It was already in 2004 that Katz found no increase in the rates of spontaneous abortions in a group of 96 infliximab-treated pregnant women with RA and Crohn’s disease compared to the general population [106,107]. Many later randomized trials, meta-analyses and systematic reviews from registries (PIANO over 1000 patients), (TREAT) assessing maternal exposure to infliximab have shown no increased risk of congenital malformations, neonatal complications, or preterm delivery [41,42,43,44,45,99,100,101,102,103,104,108].

Also, a large multicenter European TEDDY study showed no relationship between perinatal anti-TNF-α therapy and any developmental delays or abnormalities [109] in infants [110,111]. A large retrospective multicenter study in 388 children exposed to anti-TNF-α therapy also showed no increase in the short- or long-term risk of severe infections [112].

Breastfeeding while on infliximab is acceptable when the risk of treatment discontinuation would lead to maternal disease exacerbation.

Given the presence of Fc receptors, the drug is expected to be absorbed with breast milk. [113]. This was verified in a large prospective, a multicenter study assessing infliximab levels in the breast milk of infliximab-treated mothers with IBDs [114]. Low levels of the drug were detected in breast milk samples from 19/29 (66%) infliximab-treated patients. Peak levels occurred up to 24 and 48 h after infusion, however, such low drug levels did not increase the risk of infection. Positive data were also obtained from the PIANO registry, which showed no increase in infections in infants exposed to biologics during breastfeeding [42].

## 7. Adalimumab

Like infliximab, adalimumab is actively transported across the placenta by FcRn. Drug levels in both newborn blood and umbilical cord blood reflect active placental transport, which was confirmed in a small study including 10 cases after maternal exposure to adalimumab [98]. Infant levels of the drug ranged from 4.28 to 17.7 μg/mL and were higher than in all mothers using adalimumab. The levels of adalimumab were still detectable 11 months after birth.

A larger multicenter study assessed 36 women exposed to adalimumab. The median concentration in the cord blood was 2.0 µg/mL [36]. The median neonatal/maternal concentration ratio was 1.21, with the mean time to neonatal clearance of up to 4 months. As opposed to infliximab, maternal adalimumab levels were found to remain stable throughout pregnancy [29]. The umbilical cord/maternal level ratio indicated higher infant levels of infliximab compared to adalimumab [115].

Compared to infliximab, there are fewer studies assessing the efficacy of adalimumab. However, long-term studies assessing the safety of adalimumab in RA in 74 exposed pregnant women confirm the safety of this drug. This study found no difference in birth defects, premature deliveries, or spontaneous abortions compared to disease-matched controls.

As with infliximab, several cohort studies, registries, and meta-analyses also showed no increase in the risk of spontaneous abortion, birth defects, or premature delivery in pregnant women exposed to adalimumab [42,43,106,116].

Adalimumab can be also safely used during breastfeeding. The data comes from a multicenter, prospective study in which adalimumab was detected in only 2 out of 21 breast milk samples [114]. The maximum breast milk level detected was 0.71 µg/mL, and was not associated with any adverse developmental or infectious events. Another published case report assessed maternal serum and milk levels every two days after the administration of adalimumab. Notably, the level of adalimumab in breast milk was lower than 1/100 of its serum level. However, further data are important to determine whether this low level may still have local effects in the intestinal mucosa and to clarify whether it may result in an infant sensitizing or tolerating the exogenous antibody. However, further data are needed to determine if this low level could still have local effects in the intestinal mucosa, as well as to verify if it may sensitize or tolerize the infant towards the exogenous antibody [117].

## 8. Etanercept

Compared to monoclonal antibodies, only minimal amounts of etanercept cross the placenta. A study published by the European League Against Rheumatism (EULAR) in 2016 described 3 cohort studies, 3 follow-up cases, 2 data registries, and 11 case reports or series of 332 pregnancies exposed to etanercept, of which 213 were followed up prospectively. In this group, there were 16.2% of miscarriages (data did not differ from the controls) and 3.6% risk of congenital defects (again the data did not differ from the controls). To summarise, there is no evidence for an increased risk of miscarriage or congenital defects in patients treated with etanercept. Alternatively, the treatment may be continued until 30–32 weeks of gestation, and, if there is a high risk of exacerbation of the underlying disease, the treatment may be continued throughout pregnancy due to minimal placental transfer [118].

Since only negligible amounts of etanercept undergo placental transfer, and the drug has a good safety profile, the EULAR recommendations allow the use of etanercept during breastfeeding in exceptional circumstances. However, further prospective research and analyses of databases are needed.

## 9. Certolizumab

The lowest placental transfer was found for certolizumab, as confirmed by in vitro and in vivo studies [119]. The FcRn binding affinity (KD) was 132 nM, 225 nM, and 1500 nM for infliximab, adalimumab, and etanercept, respectively. There was no measurable binding affinity for certolizumab pegol, similar to that of the control group (healthy pregnant women). CZP placental transport was below detectable levels in 5 of 6 human placentas, which were positively IgG perfused ex vivo [119].

The study measured anti-TNF-α for three structural categories: monoclonal antibodies (ADA and IFX), TNF receptor fusion proteins-IgG Fc (ETA), and a monoclonal antibody Fab’ fragment (CZP). IFX and ADA monoclonal antibodies were found to have a high affinity for FcRn, which is not surprising given their intact Fc region in the structure of the drug molecule. The TNF receptor-IgG Fc fusion protein ETA, which also has an Fc region, showed a lower affinity for FcRn compared to the monoclonal antibodies, although the binding was still measurable. However, its levels were low (FcRn-mediated transcytosis across a cell layer (mean ± SD; *n* = 3) was 249.6 ± 25.0 (IFX), 159.0 ± 20.2 (ADA), 81.3 ± 13.1 ng/mL (ETA), and 3.2 ± 3.4 ng/mL (CZP)) [119]. This reduced affinity for FcRn has been reported previously (Suzuki et al.), and it may be due to the fusion of the TNF receptor to the Fc region, which either alters the conformation of the binding region or induces steric hindrance, which in turn affects FcRn binding [116].

Another study again assessed the CZP placental transfer. CRIB was a pharmacokinetic (PK) study to assess the level of CZP in women ≥30 weeks pregnant and postpartum. Blood samples were collected from mothers, umbilical cords, and infants at delivery, and CZP was measured in infants at 4 and 8 weeks post-delivery. The study showed negligible placental CZP transfer of 0.042 μg/mL (infant/mother plasma ratio 0.0009); no measurable levels of CZP were found in infants at weeks 4 and 8. The study suggests a lack of in utero fetal exposure during the third trimester. These findings support the continuation of CZP treatment throughout pregnancy when considered necessary. However, more extensive studies and meta-analyses are needed to confirm these findings [120].

A prospective analysis of the safety database report of certolizumab in 528 pregnant women showed no increased risk of congenital malformations or fetal loss compared to the general population [115].

Another aspect is the breast milk level of a biological (CZP) during breastfeeding, and drug levels in infants, which may translate into the risk of infection or vaccination with live vaccines.

The CRADLE study, which was published in 2017, was the first study to assess maternal breast milk CZP levels and the estimated Average Daily Infant Dose (ADID). CZP levels were measured after 3 doses of the drug. A total of 17 CZP-treated mothers were screened; no measurable CZP levels were detected in 77/137 (56%) breast milk samples. Samples from 4/17 mothers were below the Lower Limit of Quantification (LLOQ). Estimated ADID ranged 0–0.0104 mg/kg/day; the median Relative Infant Dose (RID) was 0.15%. The authors concluded that there was no transfer of CZP from plasma to breast milk and that the drug has a good fetal safety profile [121].

## 10. Golimumab

FcRn also actively transports golimumab across the placenta. Golimumab levels were measured in the umbilical cord blood of an infant whose mother was treated with the drug during pregnancy for ulcerative colitis. Neonatal levels were 8 µg/mL, which corresponds to 121% of maternal plasma levels at birth [110]. A review of 40 golimumab-exposed pregnant women (10 women with ulcerative colitis) reported a higher rate of spontaneous abortions (32.5%) compared to the general population [111]. Almost a third (30.8%) of these patients also received methotrexate. The analysis of these data should be done carefully. The rate of birth defects was similar to that in the general population.

There is limited evidence for the use of golimumab during pregnancy and breastfeeding. Therefore, it is recommended to switch to a drug with a known safety profile; however, the EULAR recommendations allow the use of golimumab during breastfeeding under special circumstances.

## 11. Anti- TNF-α Therapy and Family Planning—Is It Safe?

When using immunosuppressants and drugs that modify the course of rheumatic disease, the greatest concerns are raised about their impact on birth defects in a child. They also increase the risk of infections. This applies not only to classic disease-modifying drugs, such as methotrexate, leflunomide, sulfasalazine, antimalarial drugs, but also biologic disease-modifying anti-rheumatic drugs (bDMARDs), e.g., TNF-α inhibitors [122,123,124,125].

It is worth emphasizing that complete discontinuation of immunosuppressive treatment due to concerns about the child’s safety may lead to an exacerbation of rheumatic disease, which in turn may affect the course of pregnancy (e.g., premature birth, low birth weight).

Discontinuation of anti-TNF-α therapy in early pregnancy causes several problems. It is associated with an increased risk of disease exacerbation during pregnancy and puerperium, as well as an increased risk of anti-drug antibodies due to lower trough levels of the biological drug. This can lead to a loss of drug response when treatment is resumed. In the case of patients with inflammatory bowel diseases, anti-TNF-α treatment can be discontinued during pregnancy in patients at a very low risk of relapse, with objectively stable (endoscopic) remission lasting from <6 months before conception, without previous loss of response to anti-TNF-α drugs or the need for dose adjustment, appropriate therapeutic drug levels prior to conception, no hospitalization in the past 3 years, and no history of bowel resection [87].

As mentioned before, there is no evidence that continuing anti-TNF-α therapy during pregnancy has a negative effect on pregnancy or the child [126,127]. In the case of patients with active disease in the second trimester, the benefits of continuing anti-TNF-α therapy in the third trimester outweigh the potential risks [42,112]. The dose of biological agents may be adjusted to achieve minimum or trough serum levels at the time of delivery to minimize placental transfer at the time of delivery.

Summarising the collective literature data, a large meta-analysis including eight prospective studies with comparative groups was published in 2019. TNF-α inhibitors were associated with a significantly higher risk of low birth weight (odds ratio (OR) 1.43; 95% CI 1.00–2.04) and significantly lower live birth rates (OR 0.61; 95% CI 0.38–0.98). However, there were no significant differences in the risk of birth defects, therapeutic abortion, spontaneous abortion, and premature delivery between the two study groups [128].

Similar data were published for a large population-based study including women with inflammatory bowel disease, rheumatoid arthritis, ankylosing spondylitis, psoriatic arthritis, and psoriasis, and their infants born between 2006 to 2013 from the national health registers in Denmark, Finland, and Sweden. The women treated with anti-TNF-α therapy were compared to those not receiving biologicals. The following agents were evaluated: adalimumab, etanercept, and infliximab used in early pregnancy. Among 1,633,909 births, 1027 infants were born to women treated with anti-TNF and 9399 to women with nonbiologic systemic treatment. Compared with nonbiologic therapy, women treated with anti-TNF-α had a higher risk of preterm birth (odds ratio 1.61 (1.29–2.02)), and cesarean section (1.57 (1.35–1.82)). The odds ratio for small for gestational age was 1.36 (0.96–1.92) for infliximab. The risk of small for gestational age was higher for inflammatory joint and skin diseases, but not for inflammatory bowel disease. Discontinuation of anti-TNF-α had opposite effects on preterm birth due to IBD and inflammatory joint and skin diseases [129].

The presented evidence confirms that each patient should be individually assessed for the balance of risks and benefits in order to select the therapy so that it is safe for both the mother and the fetus.

## 12. Recommendations for the Management in Pregnancy-Planning Patients Treated with Anti-TNF-α Drugs Based on the Guidelines of Rheumatology and Gastroenterology Societies

### 12.1. The Period of Procreation

Effective contraception should be used during biological therapy and for an appropriate period thereafter, as recommended for individual medicinal products in their summaries of product characteristics (SmPCs). Biological anti-TNF-α drugs should be discontinued for 6 months (infliximab, golimumab), 5 months (adalimumab, certolizumab pegol), or 3 weeks (etanercept) before the planned conception.

The approach to planning children in patients receiving this type of immunosuppressive treatment changes with gaining experience and knowledge about the risk of biological therapies. All recommendations on the treatment of patients with rheumatic diseases suggest that biological drugs should be used with caution when planning a pregnancy. According to the 2016 opinion of the British Society for Rheumatology (BSR) and the British Health Professionals in Rheumatology (BHPR) [78], certolizumab, infliximab, etanercept, and adalimumab may be used in the periconceptional period in women. Golimumab does not have such recommendations due to the lack of a sufficient number of periconceptional studies [78]. On the other hand, according to the latest 2020 recommendations of the American College of Rheumatology (ACR), all anti-TNF-α drugs can be conditionally recommended in periconceptional women [77]. Certolizumab pegol is a preferred option during this period (Table 2) [77,78,130,131,132,133,134,135].

According to the 2020 recommendations of the American College of Rheumatology (ACR), anti-TNF-α therapies (adalimumab, certolizumab pegol, etanercept, golimumab, and infliximab) can be used (if needed) for the treatment of male partners prior to conception. Other biological drugs (anakinra, belimumab, abatacept, tocilizumab, secukinumab, ustekinumab) lack such a recommendation due to insufficient data on their safety [77]. According to the guidelines of the American Gastroenterological Association (AGA), pregnancy should be planned during remission, biological therapy (including anti-TNF-α) should not be discontinued during this period, and measuring the levels of the biological drug should be considered [134]. The European Crohn’s and Colitis Organization (ECCO) guidelines recommend planning a pregnancy during remission, and even discontinuation of anti-TNF-α treatment in patients in remission who plan pregnancy [105,132].

### 12.2. Pregnancy

Most biological drugs used in rheumatology are contraindicated during pregnancy and breastfeeding (as stated in their SmPCs). Certolizumab pegol and adalimumab are exceptions. The SmPC of certolizumab pegol informs that the product “should only be used during pregnancy if clinically needed”. The SmPC of adalimumab informs that “adalimumab should only be used during pregnancy if clearly needed”. Therefore, biological drugs, including anti-TNF-α agents, are discontinued in pregnancy.

In individual cases, where discontinuation of a biological drug is known to cause severe exacerbation of the underlying disease, or where the disease exacerbates already during pregnancy after treatment discontinuation, a rheumatologist can make an individual decision to include a biological drug during pregnancy, having considered the benefits and risks of continuing immunosuppressive treatment in pregnancy. According to BSR and BHPR experts (2016) [78], biological drugs from the anti-TNF-α group (etanercept, adalimumab) can be safely used in the first and second trimesters, but not in the third trimester (theoretical increase in the risk of neonatal infection). The BSR and BHPR experts strongly recommend the use of certolizumab pegol during pregnancy if needed due to rheumatic disease. The decision always belongs to the rheumatologist and is made individually for each patient, under special circumstances, when therapy discontinuation will worsen the condition of the affected patient. According to these guidelines, other drugs, e.g., golimumab, are not recommended due to the lack of literature data on their safety during this period [78].

However, according to the latest (2020) recommendations of the American College of Rheumatology (ACR), anti-TNF-α drugs (adalimumab, etanercept, golimumab, and infliximab) can be conditionally recommended during pregnancy, but treatment can be continued only in the first/second trimester. Their use should be discontinued in the third trimester, i.e., at least a few half-lives before delivery [77]. Certolizumab pegol is the most strongly recommended drug during pregnancy, regardless of the trimester and if it is needed due to the patient’s condition [131]. (Table 3) [77,78,130,131,132,133,134,135].

The 2018 APLAR (Asia Pacific League of Associations for Rheumatology) guidelines for the treatment of RA addressed the issues related to the use of targeted therapy during pregnancy. It recommends continued anti-TNF-α therapy in the first trimester [131]. Based on research data from national registries, case series, and retrospective studies from national databases, no increase in the incidence of pregnancy loss or congenital malformations was shown compared to the control group or preliminary data for anti-TNF-α drugs [78,130,131,132,133,134,135,136]. Additionally, the European League Against Rheumatism (EULAR) task force concluded that etanercept and certolizumab are relatively safe drugs that can be used throughout pregnancy due to their low placental transfer [130].

The European Crohn’s Disease and Colitis Organization (ECCO) recommends that anti-TNF-α therapy should be discontinued in the second trimester (preferably before 30 weeks gestation) when IBD patients are in stable remission [132].

The guidelines of the American Gastroenterology Association (AGA) do not recommend discontinuation of biological drugs (including anti-TNF-α) throughout pregnancy. It is recommended that the last dose of a biological drug should be determined during pregnancy before the due date to reduce placental drug transfer: 2–3 weeks before the due date for ADA (or 1–2 weeks if ADA is administered weekly) 4–6 weeks before the due date for GOLA, 6–10 weeks before the due date for INF (4–5 weeks if INF is administered every 4 weeks). However, it is possible to continue CZP treatment during pregnancy without the need to discontinue the drug before the planned delivery date [134].

### 12.3. Breastfeeding

According to the 2016 opinion of BSR/BHPR experts [78], biological drugs from the anti-TNF-α group (etanercept, adalimumab) can be used during breastfeeding; however, the literature data are limited. BSR and BHPR strongly recommend certolizumab pegol during breastfeeding if clinically needed due to rheumatic disease [78].

The 2020 recommendations of the American College of Rheumatology (ACR) indicate that all anti-TNF-α drugs are recommended for use during breastfeeding if clinically needed [77]. The same position is taken by other gastroenterological associations, i.e., ECCO, ASGHRR (Austrian Societies of Gastroenterology and Hepatology and Rheumatology and Rehabilitation), and AGA [132,133,134,135,136].

### 12.4. Vaccinations

Most vaccinations should be performed in accordance with the immunization schedule. Live vaccines are an exception. Immunization with live or live-attenuated vaccines (e.g., BCG vaccine) is not recommended for infants exposed in utero to a biological drug for at least 5 months after the last dose is administered in the pregnant mother or until 7 months of age [78]. Also, the rotavirus vaccine should not be administered to children who have been exposed in utero to a maternal biological drug (GRADE (grade of recommendation): weak recommendation, very low-quality evidence. Agreement: 97.7%) [135].

## 13. Conclusions

To conclude, biological treatment during pregnancy or breastfeeding may be used in patients with active inflammatory diseases only in individual cases and after establishing a treatment strategy, with biological therapy planned already during the procreation-planning period. This is a choice that takes into account the benefits, but also the risks associated with the continuation of immunosuppressive treatment during conception, pregnancy, and breastfeeding, therefore a consensus is needed between the patient, the rheumatologist, or gastroenterologist, and the obstetrician-gynecologist.

## Figures and Tables

**Table 1:** US Food and Drug Administration categories for drug safety during pregnancy.

FDA Category	Description
A	Adequate and well-controlled studies have failed to demonstrate a risk to the fetus during the first trimester of pregnancy (and there is no evidence of risk in the later trimesters)
B	Animal reproduction studies have not demonstrated a fetal risk, but there are no adequate and well-controlled studies in pregnant women. Or Animal reproduction studies have shown an adverse effect, but adequate and well-controlled studies in pregnant women have failed to demonstrate a risk to the fetus during the first trimester of pregnancy (and there is no evidence of a risk in the later trimester)
C	Animal reproduction studies have shown an adverse effect on the fetus, there are no adequate and well-controlled studies in humans, and the benefits from the use of the drug in pregnant women may be acceptable despite its potential risks. Or There are no animal reproduction studies and no adequate and well-controlled studies in humans.
D	There is positive evidence of human fetal risk based on adverse reaction data from investigational or marketing experience or studies in humans, but the potential benefits from the use of the drug in pregnant women may be acceptable despite its potential risks
X	Studies in animal or humans have demonstrated fetal abnormalities or there is positive evidence of fetal risk based on adverse reaction reports from investigational or marketing experience or both and the risk of the use of the drug in pregnant women

**Table 2:** TNF-alpha inhibitors management in patients with inflammatory diseases prior to conception based on the recommendations/guidelines of: (i) Rheumatological Medical Societies: British (BSR—The British Society for Rheumatology and BHPR (British Health Professionals in Rheumatology) [78]; European (EULAR—European League Against Rheumatism) [130]; Asia Pacific Region–(APLAR—Asia Pacific League of Associations for Rheumatology) [131]; American (ACR —American College of Rheumatology) [77]; (ii) Gastroenterological Medical Societies: Austrian (ASGHRR—Austrian Societies of Gastroenterology and Hepatology and Rheumatology and Rehabilitation) [133], European (ECCO—European Crohn’s and Colitis Organisation) [132], American (AGA—American Gastroenterological Association) [134].

Medical Societies Recommendations for Management of Inflammatory Diseases PRIOR TO CONCEPTION							
Society	BSR/BHPR 2016	EULAR 2016	APLAR 2018	ACR 2020	ECCO 2017	ASGHRR 2019	AGA 2019
Medication							
Adalimumab (ADA)	Can be used	No risk of congenital defects	No clear guidelines-use in pregnancy only in patients whose disease activity cannot be otherwise controlled (Grade C) *	Can be used +	No clear guidelines	Can be used	TNFi should be continued; measure serum drug levels; monotherapy as preferred option
Etanercept (ETA)	Can be used			Can be used +		Can be used	
Infliximab (INF)	Can be used			Can be used +		Can be used	
Certolizumab pegol (CZP)	Can be used			Strongly recommended ++		Can be used	
Golimumab (GOLI)	No data			Can be used +		Conditionally	

++—strong recommendation; +—conditional recommendation. *—see Table A1 (Appendix A).

**Table 3:** TNF-alpha inhibitors management in patients with inflammatory diseases during pregnancy based on the recommendations/guidelines of: (i) Rheumatological Medical Societies: British (BSR—The British Society for Rheumatology and BHPR (British Health Professionals in Rheumatology) [78]; European (EULAR—European League Against Rheumatism) [130]; Asia Pacific Region (APLAR—Asia Pacific League of Associations for Rheumatology) [131]; American (ACR—American College of Rheumatology) [77]; (ii) Gastroenterological Medical Societies: Austrian (ASGHRR- Austrian Societies of Gastroenterology and Hepatology and Rheumatology and Rehabilitation) [133], European (ECCO—European Crohn’s and Colitis Organisation) [132], American (AGA—American Gastroenterological Association) [134].

Medical Societies Recommendations for the Management of Inflammatory Diseases DURING PREGNANCY							
Society	BSR 2016	EULAR 2016	APLAR 2018	ACR 2020	ECCO 2017	ASGHRR 2019	AGA 2019
Medication							
Adalimumab (ADA)	Can be used during I and II trimester	Use throughout first half of the pregnancy (20 weeks). Throughout pregnancy- as needed	TNFi (preferred ETA and CZP) can be used throughout pregnancy in patients with established RA, if other therapeutical options are not efficient	Conditionally +; Continue in I and II trimesters, discontinue in III trimester ^#^	Treatment should be discontinued at latest week 30 or even earlier (20–25 week), if IBD is in remission Female patients in clinical remission-safe option for the mother and for the child is to discontinue TNFi in II trimester	Strong recommendation for use (LE2; GRB)	Maintain prepregnancy doses. Continue dosing throughout all 3 trimesters (CZP)If possible, plan the final dose before EDC according to the drug T1/2 to minimize transfer through the placenta: ADA—2–3 weeks: GOLI—4–6 weeks INF—6–10 weeks; base dosing on prepregnancy weight. Resume treatment not earlier than 24–48 ** h after delivery
Etanercept (ETA)	Can be used during I and II trimester	Use throughout pregnancy may be considered *.		Conditionally +; Continue in I and II trimesters, discontinue in III trimester ^#^		Strong recommendation for use (LE2; GRB)	
Infliximab (INF)	Can be used during I trimester–stop at 16 week	Use throughout first half of the pregnancy (20 weeks). Throughout pregnancy- as needed		Conditionally +; Continue in I and II trimesters, discontinue in III trimester ^#^		Strong recommendation for use (LE2; GRB)	
Certolizumab pegol (CZP)	Can be used	Use throughout pregnancy may be considered *.		Strongly recommended ++		Strong recommendation for use (LE2; GRB)	
Golimumab (GOLI)	No data	ConditionallyNo data		Conditionally + Management like ADA, ETA, INF		Conditionally limited data (LE4, G C)	

T1/2—half-life; ++ strong recommendation; + conditional recommendation; LE—Level of Evidence; GR—Grade of Recommendation. (Table A2, in Appendix A); *—due to the low index of placenta passage; ^#^—several half-lives (T1/2) prior to delivery.; IBD—inflammatory bowel disease., EDC—estimated date of confinement; **—24 h after vaginal delivery; 48 h after cesarean delivery.

## Data Availability

Data sharing not applicable.

## Appendix A

**Table A1:** Grade for quality of evidence [131,137].

Grade	Quality of Evidence	Meaning
A	High	We are very confident that the true effect lies close to that of the estimate of the effect
B	Moderate	We are moderately confident in the effect estimate. The true effect is likely to be close to the estimate of the effect, but there is a possibility that it is substantially different
C	Low	Our confidence in the effect estimate is limited. The true effect may be substantially different from the estimate of the effect
D	Very low	We have very little confidence in the effect estimate. The true effect is likely to be substantially different from the estimate of the effect

**Table A2:** (**A**) Levels of evidence (treatment benefits). (**B**) Levels of evidence (common treatment harms). (**C**) Grade of recommendation [133,138].

A	**No.**	**Levels of Evidence** (**Treatment Benefits**)
	1 *	Systematic review of randomized trials or n-of-1 trials
	2 *	Randomized trial or observational study with dramatic effect
	3 *	Non-randomized controlled cohort/follow-up study **
	4 *	Case-series, case-control studies, or historically controlled studies **
	5 *	Mechanism-based reasoning
B	**No.**	**Levels of Evidence** (**Common Treatment Harms**)
	1 *	Systematic review of randomized trials, systematic review of nested case-control studies, n-of-1 trial with the patient you are raising the question about, or observational study with dramatic effect
	2 *	Individual randomized trial or (exceptionally) observational study with dramatic effect
	3 *	Non-randomized controlled cohort/follow-up study (post-marketing surveillance) provided there are sufficient numbers to rule out a common harm. (For long-term harms, the duration of follow-up must be sufficient) **
	4 *	Case-series, case-control, or historically controlled studies **
	5 *	Mechanism-based reasoning
C	**Grade**	**Grade of Recommendation-Meaning**
	A	Consistent level 1 studies
	B	Consistent level 2 or 3 studies or extrapolations from level 1 studies
	C	Level 4 studies or extrapolations from level 2 or 3 studies
	D	Level 5 evidence is troublingly inconsistent or inconclusive in studies of any level

* Level may be graded down on the basis of study quality, imprecision, indirectness (study PICO does not match questions PICO ***), because of inconsistency between studies, or because the absolute effect size is very small; levels may be graded up if there is a large or very large effect size. ** As always, a systematic review is generally better than an individual study; *** PICO—Patient, Intervention, Comparison, Outcome.
